# Changes in the Antioxidative Activity and the Content of Phenolics and Iridoids during Fermentation and Aging of Natural Fruit Meads

**DOI:** 10.3390/biom11081113

**Published:** 2021-07-28

**Authors:** Kinga Adamenko, Joanna Kawa-Rygielska, Alicja Z. Kucharska, Adam Głowacki, Narcyz Piórecki

**Affiliations:** 1 Department of Fermentation and Cereals Technology, Wrocław University of Environmental and Life Sciences, 51-630 Wrocław, Poland; joanna.kawa-rygielska@upwr.edu.pl (J.K.-R.); adam.glowacki@upwr.edu.pl (A.G.); 2 Vegetable and Plant Nutraceutical Technology, Department of Fruit, Wrocław University of Environmental and Life Sciences, 51-630 Wrocław, Poland; alicja.kucharska@upwr.edu.pl; 3 Institute and Arboretum of Physiography in Bolestraszyce, 37-700 Przemyśl, Poland; narcyz360@gmail.com; 4 Faculty of Physical Educaiton, University of Rzeszów, 35-959 Rzeszów, Poland

**Keywords:** fermented beverages, fruit meads, natural antioxidants, phenolics, iridoids

## Abstract

The aim of the study was to investigate changes in the content of biologically active compounds during the fermentation and aging of natural meads with the addition of three Cornelian cherry juices from three cultivars: ‘Koralovyi’, ‘Podolski’ and ‘Yantarnyi’, in the amount of 10% *v*/*v*. After the fermentation process the content of gallic and ellagic acids significantly increased, in relation to wort. Whereas the greatest losses were observed among unstable anthocyanins. The three-month aging process also reduced the content of the analyzed compounds except for ellagic acid, the content of which increased by up to 90%. The content of biologically active compounds, including iridoids and antioxidant phenolics, are constantly changing in the process of fermentation and aging of fruit meads. The studies proved that the addition of Cornelian cherry juice allows significantly enriched classic meads with new biologically active compounds, such as: exceptional iridoids (loganic acid, cornuside, loganine, sweroside), flavonols, phenolic acids and anthocyanins.

## 1. Introduction

Mead is a traditional alcoholic beverage, with a real alcohol content between 9% and 18% *v*/*v*, obtained as a result of alcoholic fermentation of honey wort, with the possibility of adding alcohol, sweetening with one or more substances, adding herbs or spices or coloring, but only with burnt sugar. In the case of ‘trójniak’ type mead, one volume of honey and two volumes of water or water with fruit juice are used. Mead fermentation time depends on many factors, including the ratio of honey and water in the wort, the additives, the type of yeast strain and its condition, temperature, oxygenation of the wort, the level of the extract and the amount of nutrients in the medium. The aging of mead is an extremely important stage in which the sensory properties of this drink are formed, its stabilization and clarification. A bouquet of flavors and aromas is created. It is not uncommon for these drinks to be aged in wooden barrels in order to provide the appropriate properties. The aging time of mead depends mainly on the type of beverage and it can be aged for a few months, in the case of ‘czwórniak’, even up to 12 years in the case of ‘półtorak’.

The sale and consumption of mead increases every year not only in European countries, but also in the United States, China and other Asian countries. It has been proved that honey is a valuable raw material characterized by a high content of antioxidants [1]. Additional enrichment of mead based on bee honey with fruit raw materials with a strong antioxidant potential makes it possible to obtain products with a very high natural antioxidant content [2].

Currently, scientific research on fermented beverages is focused on the possibility of enriching products with compounds showing biological activity, having a positive effect on the human body [3]. Much research is devoted to the analysis of compounds with antioxidative properties [4]. The addition of fruit, herbs, or spices in the production technology of classical fermented beverages allows for a significant enrichment of products with natural antioxidants [5]. Moreover, it has been proved that the use of Cornelian cherry fruits gives the possibility of enriching fermented beverages not only with phenolics, but also with biologically active iridoids [6,7].

Other authors investigated changes in polyphenolic content in the production of beverages such as grape wines [8], sparkling wines [9], beer [10] or ciders [11]. Regarding meads, the literature contains research papers analyzing the composition and changes in the content of phenolic compounds under the influence of herbal or fruit additives [2] as well as under the influence of the yeast strain and heating process of the honey wort [12]. However, there are no studies available that accurately describe changes in the content of biologically active compounds, including antioxidants, during fermentation and aging of mead. Both alcoholic fermentation and aging processes are essential elements in the technology of mead production [13]. These processes are necessary not only for the production of an appropriate alcohol content in the final products, but also for the production of balanced flavor and aroma characteristics of mead, which depend on the type of raw materials, biological material, production process, time and storage conditions [14].

The production of mead requires long-term technological operations, such as fermentation or aging, as a result of which the content of biological compounds is constantly changing. The aim of the research was to analyze the changes in the composition and content of phenolics and iridoids as well as the antioxidative activity of meads produced with the addition of Cornelian cherry juice. It is important to analyze the changes of biologically active compounds that occur during the production of this type of fermented beverages to modify the process and obtain a product with the highest possible content of potential health-promoting ingredients.

## 2. Materials and Methods

### 2.1. Materials

#### 2.1.1. Reagent and Standard

6-hydroxy-2,5,7,8-tetramethylchroman-2-carboxylic acid (Trolox); 1,1-Diphenyl-2-picrylhydrazyl radical (DPPH^•^); dimethyl sulfoxide (DMSO), 2,4,6-tris(2-pyridyl)-s-triazine (TPTZ), acetonitrile, ferric chloride, sulfuric acid, formic acid, hydroxymethylfurfural (HMF), K_2_HPO_3_, CaCO_3_ and sodium hydroxide were purchased from Sigma-Aldrich (Steinheim, Germany). Acetonitrile for HPLC was purchased from POCh (Gliwice, Poland). Acetic acid was acquired from Chempur (Piekary Sląskie, Poland). Whereas loganin (Lo), loganic acid (LA), gallic acid (GA), sweroside (S), ellagic acid (EA), *p*-coumaric acid (*p*-CuA), 5-*O*-caffeoylquinic acid (5-CQA, chlorogenic acid), kaempferol 3-*O*-glucoside (Kf glc), cyanidin 3-*O*-glucoside (Cy glc) and quercetin 3-*O*-glucoside (Q glc) were purchased from Extrasynthese (Lyon Nord, France). All reagents were of analytical grade.

#### 2.1.2. Material

Rapeseed honey was purchased from Miody Polskie (Miedźno, Poland). The product came from a Polish apiary located in ecologically clean areas. According to the producer’s declaration, rapeseed honey was based solely on the production by healthy bees and did not contain any additives. The producer tested rapeseed honey in the laboratory for its prohealth properties and ecological purity. Cornelian cherry juices were used for the production of fruit meads. Juices from 3 cultivars of fruit were used: ‘Koralovyi’, ‘Podolski’ and ‘Yantarnyi’: coral, red and yellow, respectively. The fruit were collected from the Arboretum and Institute of Physiography in Bolestraszyce (49°49′01.1″ N, 22°51′24.2″ E). Fruits were pressed through the Zodiak laboratory hydraulic press from SRSE company (Warszawa, Poland).

#### 2.1.3. Microorganisms

The biological materials used for the alcoholic fermentation of honey and honey fruit worts were *Saccharomyces bayanus* Safspirit FD-33 yeast from the Fermentis by Lesaffre company (Lesaffre, Marcq-en-Barœul, France). It is recommended for fruit fermentation due to it fructophilic character, reported as a neutral strain, but in some cases produces refined and balanced esters and low nitrogen demand and high resistance to alcohol. Before inoculation, dried yeast was rehydrated in distilled and sterilized water at a temperature of 25 °C for 20 min. According to the manufacturer’s declaration, the selected yeast strain is characterized by high resistance to ethyl alcohol and is dedicated to the fermentation of fruit worts.

### 2.2. Methods

#### 2.2.1. Experimental Design

According to the production technology of the ‘trójniak’ type mead, rapeseed honey was combined with water at a 1:2 volume ratio. The obtained honey wort was subjected to a boiling process at 100 °C for 45 min. During this step, the wort was regularly mixed and the precipitating scum was removed. The broth was then cooled to 20 °C and the extract was adjusted to 34˚Bx. The obtained wort was divided into 4 types: classical honey wort (0W) and three honey fruit wort to which 10% Cornelian cherry juice of selected varieties was added: yellow juice ‘Yantarnyi’ variety (JW), coral juice ‘Koralowyi’ variety (KW) and red juice ‘Podolski’ variety (PW). The prepared wort was inoculated with yeast in the amount of 0.5 g d.m./L. Mineral salts in the form of potassium phosphate dibasic (0.4 g/L) and calcium carbonate (0.4 g/L) were also added to the fermentation medium. The fermentation settings were shaken manually in the first hours and days of fermentation in order to aerate the wort. Ethanolic fermentation was carried out for 14 days at the temperature of 22 °C, and then aged for 3 months at the temperature of 8 °C. All experimental variants were prepared in triplicate.

#### 2.2.2. Analytical Methods

##### Total Polyphenolic Content

The total polyphenolic content was determined using the Folin–Ciocalteu (F-C) spectrophotometric method described and modified by Prior, Wu and Schaich (2005) [15]. Meads samples in the amounts of 0.1 mL, 0.9 mL of ethanol and 0.2 mL F-C reagent were pipetted into cuvettes. After 3 min, 1 mL of a 20% aqueous solution of sodium carbonate (Na_2_CO_3_) and 2 mL of distilled water were added. Thereafter, all cuvettes were placed in the darkroom for 60 min. The absorbance at 765 nm was measured after 1 h, and the results were expressed as mg of gallic acid equivalents (GAE) per 1 L of mead (mg GAE/L). Data were expressed as the mean value of three measurements. Folin–Ciocalteu does not only quantify the total polyphenols of the samples, other chemical compounds can also be estimated by the reduction capacity of Folin–Ciocalteu. Therefore, the results obtained with this method should be considered very generally.

##### Free-Radical-Scavenging Ability Using a DPPH Radical

Antiradical activity was determined using DPPH^•^ assay [16]. Furthermore, 0.1 mL samples of meads were mixed with 2 mL of 0.04 mmol/L DPPH^•^ ethanolic solution and 0.4 mL of distilled water. After 10 min of incubation at room temperature, the absorbance was measured with a spectrophotometer at 517 nm using disposable polystyrene cuvettes.

##### Free-Radical-Scavenging Ability Using ABTS Radical Cation

The antioxidative activity of meads was determined using the ABTS^•+^ assay [17]. The ABTS^•+^ solution was diluted with an absorbance of 0.700 ± 0.040 at 734 nm. Furthermore, 0.06 mL samples of mead were added into cuvettes with 3 mL of ABTS^•+^ solution. The cuvettes were then left at room temperature for 6 min. After this time, the absorbance of the samples was measured at a wavelength of 734 nm.

##### Ferric Reducing/Antioxidant Power (FRAP) Assay

A FRAP method is based on the reduction of ferric 2,4,6-tris(2-pyridyl)-1,3,5-triazine (Fe(III)-TPTZ) to the ferrous complex at low pH, followed by spectrophotometric analysis [18]. Briefly, the reagent was prepared by mixing 10 mmol 2,4,6-Tris(2-pyridyl)-s-triazine (TPTZ)/L reagent with 20 mmol/L ferric chloride in acetate buffer (pH 3.6). Furthermore, 0.1 mL samples of mead were mixed in polystyrene cuvettes with 0.9 mL of distilled water and 3 mL of ferric complex. The mixture was shaken and left at room temperature for 10 min. The absorbance was read at 593 nm after 10 min.

All UV–Vis measurements were recorded on a Shimadzu UV–2401PC (Kyoto, Japan). All the determinations were performed in triplicates. Results of antioxidant capacity were expressed in mmol Trolox equivalent (TE) per liter of mead (mmol TE/L).

##### Determination of Phenolics and Iridoids Content by HPLC-PDA

The HPLC-PDA analysis was conducted by using a Dionex (Germering, Germany) system equipped with the diode array detector model Ultimate 3000, quaternary pump LPG-3400A, autosampler EWPS-3000SI, thermostated column compartment TCC-3000SD, and controlled by Chromeleon v.6.8 software (Thermo Scientific Dionex, Sunnyvale, CA, USA). The Cadenza Imtakt column CD-C18 (75 × 4.6 mm, 5 µm) column (Imtakt, Kyoto, Japan) with guard column Cadenza (5 × 4.6 mm, 5 µm) was used. The mobile phase was composed of solvent A (4.5% aq. formic acid, *v*/*v*) and solvent B (100% acetonitrile). The elution system was as follows: 0–1 min 5% B in A, 1–20 min 25% B in A, 20–26 min 100% B and 26–30 min 5% B in A. The flow rate of the mobile phase was 1.0 mL/min and the injection volume was 20 µL. The column was operated at 30 °C. Iridoids were detected at 245 nm, phenolic acid at 320 and 280 nm, flavonols at 360 nm, anthocyanins at 520 nm and HMF at 280 nm. Chromatograms of analyzed compounds (Figures S1–S5) in fruit meads are included in the Supplementary Materials section. Calibration curves at concentrations ranging from 0.01 to 0.30 mg/mL were mode for loganic, gallic, ellagic, *p*-coumaric and 5-*O*-caffeoylquinic acids, loganin, quercetin 3-*O*-glucoside, kaempferol 3-*O*-glucoside and hydroxymethylfurfural as standards. Additionally, derivatives of gallic, ellagic and *p*-coumaric acids were expressed as gallic, ellagic and *p*-coumaric acids, respectively, and cornuside was expressed as loganic acid, sweroside as loganin, quercetin 3-*O*-glucuronide as quercetin 3-*O*-glucoside and kaempferol derivatives were expressed as kaempferol 3-*O*-glucoside. Identification (UPLC retention times, spectra UV/Vis and MS) of compounds of cornelian cherry juice by LC-MS as Table S1 is included in the Supplementary Materials section. The results were expressed as mg per 100 mL of mead.

##### Determination of Extract and pH

Extract content (% *w*/*w*) was measured with a Densito 30 PX Density Meter produced by Mettler-Toledo company (Greifensee, Switzerland). The wort and meads samples were centrifuged before measuring the extract using an MPW-351R laboratory centrifuge (2675 centrifugal force (RCF), 6000 rpm, 10 min) by MPW MED. INSTRUMENTS company (Warszawa, Poland). Extract content was measured in the resultant supernatant at a temperature of 20 °C. The pH value was analyzed with an MP 220 pH meter by Mettler Toledo company (Greifensee, Switzerland).

##### Carbohydrates, Ethanol, Glycerol, and Acetic Acid Content

The concentrations of carbohydrates, ethanol, glycerol and acetic acid were determined by means of HPLC. Degassed and centrifuged (2675 centrifugal force (RCF), 6000 rpm, 10 min) samples were diluted with ultrapure water in the volumetric ratio of 1:9; then filtered through a nylon syringe filter with a pore size of 0.22 μm into chromatographic vials. The samples were analyzed using a Prominence liquid chromatography system (Shimadzu Corp., Kyoto, Japan) equipped with a Rezed ROA-Organic Acid H+ column (300 × 4.6 mm) from Phenomenex (Torrance, CA, USA). The following parameters of measurements (ethanol, glycerol and acetic acid) were applied: injection volume 20 μL, elution temperature 60 °C, flow rate 0.6 mL/min, mobile phase 0.005 M H_2_SO_4_ and thermostat refractometric detector at 50 °C. Whereas parameters for measurements of carbohydrates were: injection volume 20 μL, elution temperature 50 °C, flow rate 0.4 mL/min, mobile phase 0.005 M H_2_SO_4_ and thermostat refractometric detector at 50 °C. Concentrations of ethanol, carbohydrates, glycerol and acetic acid were determined based on a five-point calibration curve integrated in Chromax 10.0 software by Pol-Lab (Wilkowice, Poland).

##### Statistical Analysis

Mean deviations are shown in the tables and in the figures. Selected data were processed using Statistica 13.5 software (StatSoft, Tulsa, OK, USA) for calculating one-factor analysis of variance (ANOVA) with a significance level α = 0.05. Differences between means were tested with the Duncan test (*p* < 0.05).

## 3. Results and Discussion

### 3.1. Total Polyphenols Content and Antioxidative Activity

**Figure 1:**
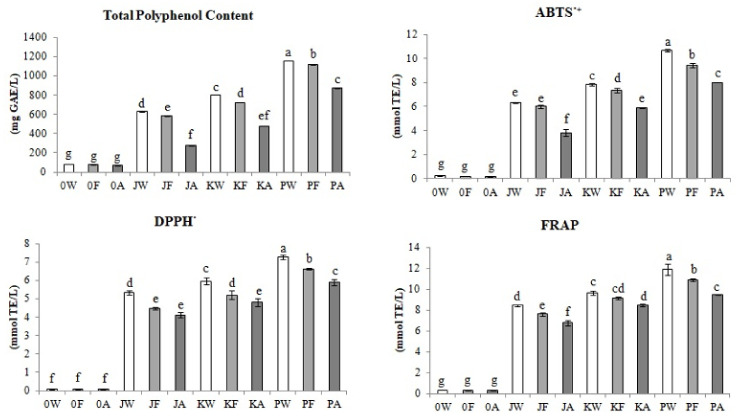
Changes in total polyphenols content (mg GAE/L) and antioxidative activity (mmol TE/L) measured by ABTS^·+^, DPPH^·^ and FRAP tests during control and fruit meads production technology. White bars—worts (W), light gray bars—samples after fermentation (F), dark gray bars—meads after aging (A). Sample codes: 0—control, J—samples with ‘Jantarnyi’ juice, K—samples with ‘Koralowyi’ juice, P—samples with ‘Podolski’ juice. Mean values with different letters: a, b, c, d, e, f are statistically different (*p* < 0.05).

Figure 1 shows changes in the total content of polyphenols and antioxidative activity during the meads production technology.

It was proved that the addition of Cornelian cherry juice significantly increases the content of polyphenols in final meads. The highest content of total polyphenols was measured in mead with juice from Cornelian cherry fruit of the ‘Podolski’ cultivar (PA). The content of polyphenols in this sample—896.60 mg GAE/L—was over 13 times higher than that of the classic control mead (0A)—66.60 mg GAE/L. In the alcohol fermentation cycle, the total polyphenols content in the control samples decreased by 3.5% (0F), and as a result of aging by 15% (0A). The entire process of classical mead production technology reduced the content of polyphenols by 18%. As a result of the fermentation process, fruit meads lost from 3.5% of total polyphenols with Cornelian cherry juice from the fruit of the ‘Podolski’ (PF) cultivar to 10% with juice from the fruit of the ‘Koralovyi’ cultivar (KF). Aging reduced polyphenols in the final products from 22% in the mead with juice from the fruit of the ‘Podolski’ cultivar (PA) to 53% in the mead with juice from fruit of the ‘Jantarnyi’ cultivar (JA). The entire technological process decreased the content of polyphenols in fruit meads from 25% in the PA sample to 56% in the JA sample.

The results of the antioxidative activity of the tested meads correctly correlated with the results of the total content of polyphenols. On the basis of the obtained results, it was proved that the highest antioxidative activity (measured with ABTS·^+^ test) was found in PA mead, 8.02 mmol TE/L, the value of which was more than 50 times higher than in the control sample without Cornelian cherry juice (0A)—0.16 mmol TE/L. In the ABTS·+ test, the antioxidant activity of control meads decreased by 21% (0F) as a result of alcoholic fermentation, and by 16% (0A) as a result of the aging process. As a result of the whole process, the antioxidant activity of classic mead decreased by 33%. In the case of fruit mead, the antioxidant activity after fermentation decreased from 5% in the variant with fruit juice of the ‘Jantarnyi’ (JF) cultivar to 12% in the sample with the fruit juice of the ‘Podolski’ (PF) cultivar. On the other hand, the aging process caused a decrease in the antioxidative activity from 25% in KA and PA meads to 40% in the JA sample.

The DPPH˙ test also indicated that the addition of Cornelian cherry juices significantly increased the antioxidant potential of final products. The highest antioxidative activity measured by the DPPH^·^ test was found in the mead with juice from the fruit of the ‘Podolski’ cultivar (PA) at the level of 5.90 mmol TE/L, which was over 98 times higher in relation to the classic control mead (0A)—0.06 mmol TE/L. The antioxidative activity of classic mead measured by the DPPH^·^ test decreased by 14% as a result of the fermentation process (0F). On the other hand, the aging process did not change statistically significant the antioxidative activity of the final product (0A). The DPPH^·^ test showed that both alcoholic fermentation and aging had the effect of reducing the antioxidative activity of fruit mead, which decreased from 8.5% in the PF sample to 16% in the JF sample after the fermentation process. However, after the aging stage, the antioxidant potential of Cornelian cherry meads decreased from 8% in JA and KA meads to 11% in PA mead. In summary, the fruit mead production process reduced the antioxidant capacity from 18.5% in the PA sample to 23% in the JA sample.

As in the case of other analytical methods, the FRAP test showed that the strongest reductive activity was characteristic of meads with fruit juice of the ‘Podolski’ cultivar PA, at the level of 9.47 mmol TE/L, which was over 32 times higher than the control mead 0A, without the addition of fruit juice—0.35 mmol TE/L. Using the FRAP test, it was shown that, both in the control and in the fruit meads, the production technology resulted in a decrease of the reductive potential of the final products. In control samples, the reductive activity decreased after fermentation by 6% (0F), and after the aging process by 12% (0A). As a result of the entire production process, the tested capacity of the final product decreased by 17%. After the fermentation of fruit mead, the lowest 5% decrease in reductive activity was observed in the sample with the addition of ‘Koralovyi’ (KF) fruit juice, while the highest with ‘Podolski’ (PF) fruit juice—by 20%. Similarly, tendencies were observed during aging, the lowest decrease in antioxidative activity by 7.5% was found in KA mead, while the highest—by 13%—in PA mead.

Both the chemical composition and the antioxidative potential of mead depend on the pigment content in the honey and additives, on the type of additives, which most often include herbs or fruit, as well as on the mead processing [19,20]. Fermented beverages based on raw materials with a high content of polyphenols can be a very good source of natural antioxidants, because the drinks are directly absorbed into intestinal fluids, thanks to which their digestibility by the body is higher than that of food products [21]. In the available scientific literature, the authors of the study investigated the total content of polyphenols and the antioxidant activity of classic and fruit meads [12,19,21]. Socha et al. (2015) [21] in their research on fruit meads showed that the highest total content of polyphenols, which was also tested using the FC reagent method, was characteristic of a beverage with root and herb extract, however, it contained about 12 times lower polyphenols compared to the PA mead obtained in our experiment, with the juice of red fruits of the ‘Podolski’ cultivar. In turn, the antioxidant potential of meads obtained by Czabaj et al. (2017) [12] measured with the ABTS^+^ test was the highest for honeydew mead and amounted to 2.2 mmol TE/L, which was more than 3.5 times lower than the antioxidative activity of the PA mead, measured with the same method in our study. Although honey is a rich source of antioxidants [22], in the production of mead as a result of thermal treatment of wort, the content of phenolic compounds in the final products decreases significantly [12,20]. To maintain the high antioxidant activity of these types of beverages, it is important to use additives of natural herbs, spices or fruit after the wort preparation stage [12]. In studies on the influence of individual stages of the beer production process on the antioxidant activity, it was shown that, similarly to our experiments, after the fermentation process, the potential to reduce Fe^3+^ analyzed with the FRAP test was reduced by 18%. In turn, the antioxidative activity measured by the ABTS^+^ test increased by 14%. Probably, the scientific explanation of the phenomenon of the increase in antioxidant activity after fermentation is the production of reduced NADH during the conversion of glucose to pyruvate, which is a by-product of this reaction [23]. In the available scientific literature, no studies on the usefulness of FRAP or ABTS^+^ tests for the analysis of the antioxidative activity of NADH reaction systems have been found. According to Szwajgier et al. (2015) [24], the content of phenolic compounds influencing the antioxidative potential in beers decreased after the fermentation process, which could be caused by absorption by yeast and precipitation of tannins and other phenolic compounds from wort and beer.

### 3.2. Phenolics and Iridoids

#### 3.2.1. Phenolic Acids

**Figure 2:**
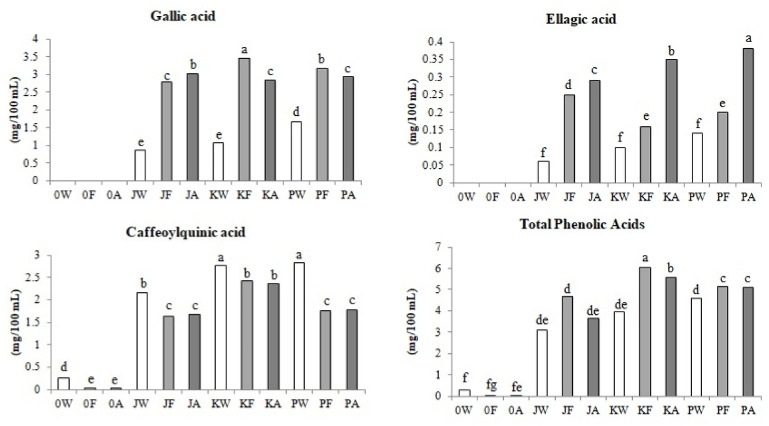
Changes in phenolic acids content (mg/100 mL) during control and fruit mead production technology. White bars—worts (W), light gray bars—samples after fermentation (F), dark gray bars—meads after aging (A). Sample codes: 0—control, J—samples with ‘Jantarnyi’ juice, K—samples with ‘Koralowyi’ juice, P—samples with ‘Podolski’ juice. Mean values with different letters: a, b, c, d, e, f are statistically different (*p* < 0.05).

Figure 2 presents changes in the content of identified and analyzed phenolic acids during the various stages of mead production.

In fruit meads: gallic, ellagic and caffeoylquinic acids and their derivatives have been identified. However, in classical meads only caffeoylquinic acids were identified. It has been proven that the addition of Cornelian cherry juice in the production of mead increased the content of phenolic acids in final products by up to 50 times. The results of the research showed that, regardless of the juice from the selected cultivar of Cornelian cherry fruit, after the fermentation stage, the content of gallic acid in the meads increased from 93% in the PF sample to 120% in the JF and KF samples. The content of gallic acid increased, probably as a result of hydrolysis of the high-molecular tannins contained in the Cornelian cherry [25]. An upward trend in the content of gallic acid by 8% was observed during the aging process of the JA mead. The inverse relationship was investigated in KA and PA meads, where after aging, the content of gallic acid decreased from 8% in the PA variant to 18% in the KA variant. The technology of the production of Cornelian cherry meads resulted in an increase the content of gallic acid in the final products from 65% in the KA mead to 150% in the JA mead. Therefore, the highest content of these compounds was found in mead with the juice of the ‘Jantarnyi’ (JA) cultivar fruit.

It was shown that both the alcoholic fermentation and the three-month aging period of fruit meads increased the content of ellagic acid in analyzed fermented beverages. Ellagic acid could also be released after the hydrolysis of large-molecule tannins contained in Cornelian cherries [25]. During fermentation, the content of ellagic acid increased from 42% in the PF sample to 215% in the JF sample. The lowest increase in the content after the treatment step was observed in the JA sample and it was 16%, while the highest was in the KA sample, by 120%; ellagic acid content by 171% was found in PA mead, while the highest—by 383%—in JA mead. Although the lowest increase in the content of ellagic acid during the production process was observed in the mead with juice from the fruit of the ‘Podolski’ cultivar, it was characterized by the highest content of ellagic acid due to the highest initial content in the wort before fermentation (PA).

The total caffeoylquinic acids consisted of: chlorogenic acid, *p*-coumaric acid and its two derivatives. In the control variant, which was classic mead, without the addition of fruit (A0), only trace amounts of *p*-coumaric acid were identified. Classical honey wort also contained low content of chlorogenic acid—at the level of 0.20 mg/100 mL (AW). The fermentation and aging process of classic meads reduced the content of caffeoylquinic acids in the final product by as much as 92%, therefore only trace amounts of the determined compounds were identified in the A0 mead. Cornelian cherry meads contained even more than 20 times higher amounts of caffeoylquinic acids (KA). It has been proven that ethanolic fermentation reduced the content of the described compounds from 12% in the KF sample to 43% in the PF sample. The aging process had no influence of the content of caffeoylquinic acids in the tested beverages. After the entire production technology of meads with Cornelian cherry juice the content of caffeoylquinic acids decreased in fermented beverages from 16.5% in KA mead to 37% in PA mead. Accordingly, the mead with juice from the fruit of the ‘Koralovyi’ cultivar (KA) was characterized by the highest total content of identified caffeoylquinic acids.

Taking into account the sum of all identified and quantified phenolic acids, in all Cornelian cherry meads, an increase in the content of these compounds after alcoholic fermentation was observed, from 11.5% in the PF sample to an average of 52% in the JF and KF samples. In turn, after the aging process, it was shown that in all fruit meads the content of phenolic acids decreased in the JA variant by 22%. As a result of the entire production process, an increase in the content of phenolic acids in meads was noted from 10% with juice from the fruit of the ‘Podolski’ cultivar (PA) to 41% with juice from the fruit of the ‘Koralovyi’ cultivar (KA). The latter, at the same time, was characterized by the highest total content of all identified phenolic acids.

#### 3.2.2. Flavonols

**Figure 3:**
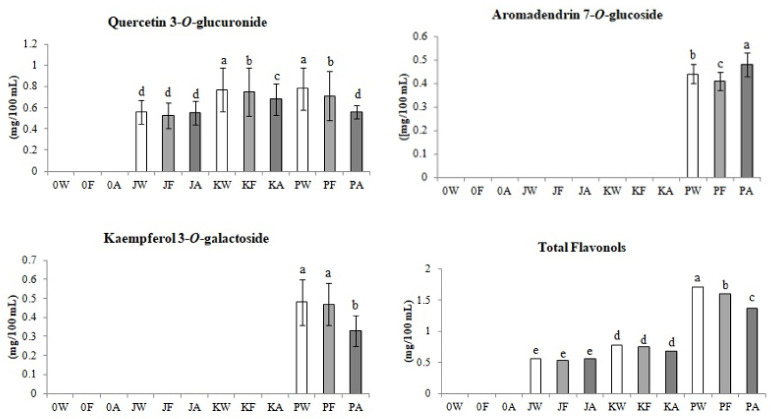
Changes in flavonols content (mg/100 mL) during control and fruit meads production technology. White bars—worts (W), light gray bars—samples after fermentation (F), dark gray bars—meads after aging (A). Sample codes: 0—control, J—samples with ‘Jantarnyi’ juice, K—samples with ‘Koralowyi’ juice, P—samples with ‘Podolski’ juice. Mean values with different letters: a, b, c, d, e are statistically different (*p* < 0.05).

Flavonols were identified only in meads, in the production of which Cornelian cherry juices were used (Figure 3).

Moreover, kaempferol 3-*O*-galactoside and aromadendrin 7-*O*-glucoside were determined only in wort and meads supplemented with red fruit juice of the ‘Podolski’ cultivar (PW, PF, PA). The highest content 1.4 mg/100 mL of all identified flavonols was found in mentioned mead (PA). Analysis of quercetin 3-*O*-glucuronide showed that after the alcoholic fermentation and aging of the JW honey-fruit wort, the content of this compound had not change significantly. On the other hand, in the case of beverages fermented with coral (K) and red (P) fruit juice, a downward trend was observed in the content of quercetin 3-*O*-glucuronide both after the fermentation and aging stages. As a result of the fermentation of the KW wort, the content of this compound decreased by 2.5%, and after the aging process by another 9% (KA). A higher decrease in the content of this flavonol, by 9%, was observed in the fermentation of the PW sample. The PA mead aging process reduced the content of the discussed compound by 21%. To sum up, the entire production process of KA and PA mead decreased the content of quercetin 3-*O*-glucuronide in the final products by 12% and 28%, respectively.

The content of kaempferol 3-*O*-galactoside did not change significantly after fermentation of the PW wort. However, after the 3-month aging stage of the mead, the content of this compound decreased by 31%. As a result of the entire production process of PA, the content of the indicated compounds decreased by 30% in relation to the wort before the alcoholic fermentation.

Aromadendrin 7-*O*-glucoside has also been identified only in meads produced with the addition of red fruit juice (PA). The fermentation of honey fruit wort (PW) reduced the content of this flavonol by 7%, while the aging of young mead by 14.5%. The overall production process of this fermented beverage led to a 9% increase of aromadendrin 7-*O*-glucoside in the final product. It has been proven that the process of fermentation and aging of PW resulted in a 20% decrease in flavonols content in the final product (PA).

#### 3.2.3. Anthocyanins

**Figure 4:**
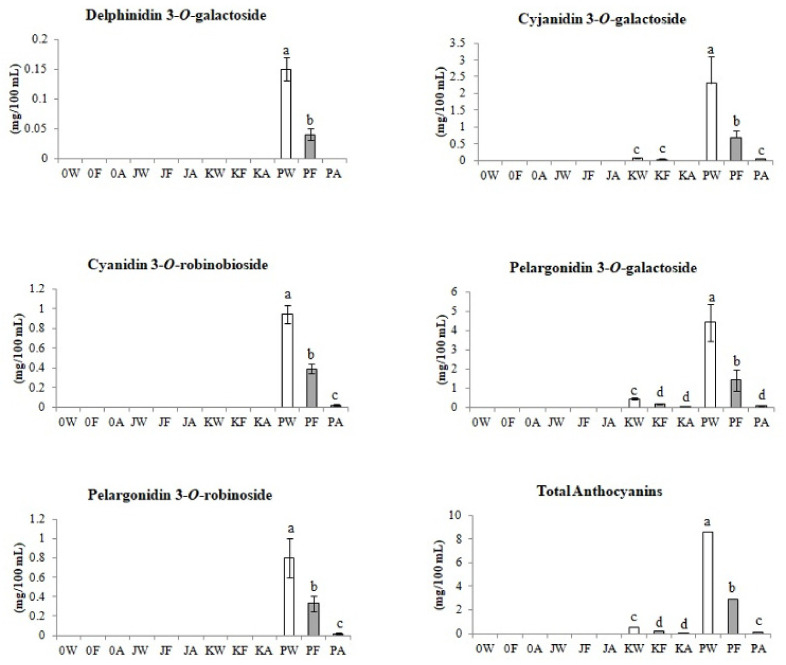
Changes in anthocyanins content (mg/100 mL) during control and fruit meads production technology. White bars—worts (W), light gray bars—samples after fermentation (F), dark gray bars—meads after aging (A). Sample codes: 0—control, J—samples with ‘Jantarnyi’ juice, K—samples with ‘Koralowyi’ juice, P—samples with ‘Podolski’ juice. Mean values with different letters: a, b, c, d are statistically different (*p* < 0.05).

As shown in Figure 4, anthocyanins were not identified in the control (classic) meads without Cornelian cherry juice (0A) and in meads with juice from the fruit of the yellow Cornelian cherry cultivar ‘Jantarnyi’ (JA).

Two anthocyanins were identified in the product with the addition of coral fruit juice ‘Koralovyi’ cultivar: cyanidin 3-*O*-galactoside and pelargonidin 3-*O*-galactoside. All five identified anthocyanins were indicated only in samples with the juice of fruits of the red colored ‘Podolski’ cultivar.

Anthocyanins were identified in the honey-Cornelian cherry wort with ‘Podolski’ juice PW at the level of 8.5 mg/100 mL. The analysis of changes in the content of anthocyanins as a result of fermentation and the 3-month aging stage of the tested meads showed that most of the anthocyanins were degraded due to the fact that anthocyanins are the most unstable group of all analyzed phenolic compounds. Therefore, only traces or no amounts of anthocyanins have been identified in the final meads.

#### 3.2.4. Iridoids

**Figure 5:**
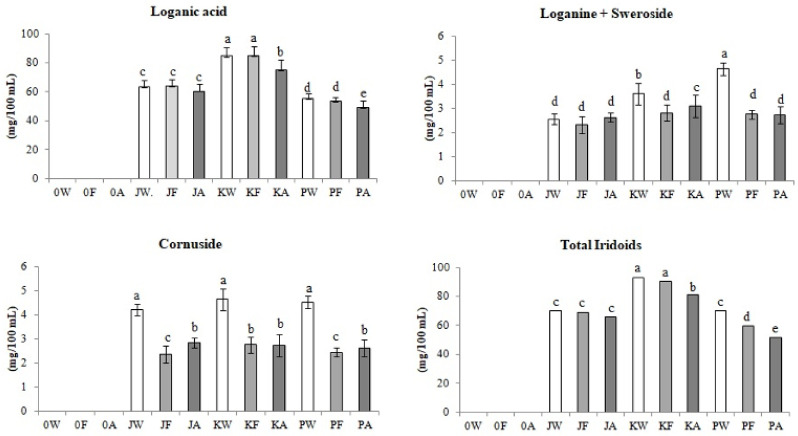
Changes in iridoids content (mg/100 mL) during control and fruit meads production technology. White bars—worts (W), light gray bars—samples after fermentation (F), dark gray bars—meads after aging (A). Sample codes: 0—control, J—samples with ‘Jantarnyi’ juice, K—samples with ‘Koralowyi’ juice, P—samples with ‘Podolski’ juice. Mean values with different letters: a, b, c, d, e are statistically different (*p* < 0.05).

Figure 5 shows changes in the content of the four identified iridoids during fermentation and aging of fruit meads.

Compounds from this group were not identified in the control meads (0A) obtained by fermentation of classic honey wort (0W). The highest total content of identified iridoids, at the level of 81.1 mg/100 mL, was found in mead with the juice of the fruit of the ‘Koralovyi’ (KA) cultivar. Loganic acid was the most dominant of the identified iridoids. It was shown that, as a result of alcoholic fermentation of JW and KW wort, the content of loganic acid in young meads only increased by 0.5–1% but this increase was not statistically significant. The opposite tendency was observed during the fermentation of the PW wort, where the content of loganic acid in young mead decreased by 4% (PF). Regardless of the type of Cornelian cherry juice in all research variants, after the aging process, the level of this acid in the finished meads decreased from 5.5% in the JA product to 11.5% in the KA production. The entire mead production process resulted in a decrease in loganic acid content from 4.5% in the JA variant to 11% in the KA and PA variants. Loganic acid constituted the largest part of all indicated iridoids but also of all biologically active compounds determined in the study. The highest content of this compound, at the level of 75.3 mg/100 mL, was found in mead with the addition of the juice of the ‘Koralovyi’ (KA) cultivar.

As a result of the experiment, it was proved that during the alcoholic fermentation of honey Cornelian cherry wort, there was a reduction in the content of loganine and sweroside in young meads from 9% in the JF sample to as much as 61% in the PF sample. An interesting change in the content of these compounds was investigated during the aging of mead with juice from the ‘Jantarnyi’ and ‘Koralovyi’ fruit varieties. Their content increased by 12% (JA) and 10% (KA), respectively. In the product with fruit juice of the ‘Podolski’ cultivar, the content of these compounds remained at the same level. Summing up the entire production process, the content of loganine and sweroside increased by 3% in the JA mead, while it decreased in KA and PA meads by 14% and 74%, respectively.

The reduction of the cornuside content after the fermentation of the honey fruit wort was similar for all variants and ranged from 40% for the KF sample to 46% for the PF sample. The content of some iridoids compounds, including cornuside, is reduced after fermentation, which may be due to the activity of yeast, which may be involved in the conversion of these compounds to bioactive derivatives [26]. A nonsignificant decrease in the content of this compound, as a result of the aging stage, was recorded in mead with coral juice and was less than 1%. In other samples, the content of this compound during the aging process increased from 6.5% in the PA mead to 17% in the JA mead. Perhaps because during aging, cornuside is released from high-molecular tannins like other iridoids in Cornelian cherry [25], or the released gallic acid and loganine from high-molecular tannins connect to form cornuside, however this has not been confirmed in the scientific literature yet. Despite the fact that in some of the tested samples, the content of cornuside increased during aging, the entire technological process resulted in the reduction of the content of this compound in the final products from 32% in JA and PA to 41% in KA.

Analysis of total iridoids showed that the content of compounds of this group, as a result of the alcoholic fermentation of honey Cornelian cherry wort, decreased from 2% in variants with yellow fruit juice (JF) to 14.5% in variants with red fruit juice (PA). The aging stage also reduced the content of identified iridoids in the final products, from 4% in JA mead to 12% in KA mead. The conducted research proved that the comprehensive process of Cornelian cherry mead production, which consisted of ethanolic fermentation and aging, led to a reduction in the iridoids content in the final products from 6% in the JA sample to 26% in the PA sample.

The type of phenolic compounds identified in meads depends on the type of honey used to prepare the wort, but also on the type of additives, where the most valuable are fruits, herbs and spices [19]. Obtained meads in this study had their source from both rapeseed honey and Cornelian cherry fruit. Rapeseed honey is a 75% source of phenolic acids and 25% source of flavonoids [27]. Whereas Cornelian cherry fruits are a good source of not only flavonols and phenolic acids, but also tannins and vitamin C [28]. Phenolic compounds have a significant impact on the quality and physico-chemical properties of food products, providing them not only antioxidative potential, but also color, taste, astringency, and extend their storage time [29].

The content of phenolic and other biologically active compounds in fruit products depends not only on the cultivar of the fruit but also on their processing and pretreatment [30,31]. The significant reduction in the content of anthocyanins after the mead fermentation and aging processes could result from the fact that anthocyanins are characterized by a very low stability due to their sensitivity to changes in pH value, time, temperature or light [32].

Iridoids are an interesting group of compounds occurring naturally in plant raw materials, and their presence, apart from cornelian fruits, has so far been detected in a small group of fruits such as: blue honeysuckle, cranberry, lingonberry or Japanese cornel [30,33]. This is a group of compounds that can impart new sensory properties to food, such as a bitter taste, i.e., oleuropein in olive oil. Despite the fact that there are open-ring iridoids in Cornelian cherry fruit, such as secologanine or cornuside, they are characteristically bitter, however, it is poorly palpable due to the low concentration of these compounds [34]. In addition, iridoids also have another important function in food. Although they do not have antioxidant potential, they are characterized by favorable biological activity, demonstrating a beneficial effect on human health. They exhibit antiplatelet, antidiabetic, cyto-, hepato-, neuro- and renal-protection, anti-inflammatory, anti-atherosclerotic, antiglaucomic activities and antimicrobial characteristics [35,36,37].

### 3.3. Basic Physicochemical Parameters

The study showed that Cornelian cherry meads were characterized by a higher content of glucose and fructose with an average value of 19.0 g/L and 13.5 g/L, respectively, compared to the control meads (Table 1).

During the fermentation of the control meads, the glucose content decreased by 88%, while for the Cornelian cherry meads by an average of 81%. As a result of aging, the reduction of glucose content in control meads was at the level of 65%, while in fruit meads from 8.5% in JA and KA samples to 14% in the PA sample. In turn, the content of fructose after alcoholic fermentation decreased by 52% in classic meads to an average of 32% in Cornelian cherry meads. After the aging process, the level of fructose in 0A decreased by another 57%, while only by an average of 3% in meads with the addition of Cornelian cherry juice.

It has been proved that classical meads contained on average 9% higher ethyl alcohol content in comparison to meads with the addition of Cornelian cherry juice. This could be due to the fact that the Cornelian cherry juice significantly increased the acidity of the wort, and the low pH value significantly reduces the fermentation activity of yeast. Moreover, the iridoids contained in Cornelian cherry fruits have antimicrobial activity, possibly also against yeast cells [37]. The three-month aging stage of young meads allowed for an increase in the alcohol content from 11% in the KA variant to 19% in the 0A and PA variants. The research proved that fruit meads had a higher glycerol content than classic meads by an average of 13%. Regarding young meads, during the aging process, the content of glycerol in all tested variants increased from 6.5% in 0A mead to 18% in the JA mead. The results of the analysis of acetic acid content showed that the Cornelian cherry meads were characterized by an average of 21% lower acetic acid content than the classic meads without the addition of fruit. Moreover, no increase in the acetic acid content in the final products was observed after the aging step of the control meads. However, in Cornelian cherry meads, the content of this acid after aging increased from 4% in the JA sample to 9% in the PA sample. Fruit meads were characterized by about three times higher content of HMF than the control meads. Cornelian cherry juice significantly increased the acidity of honey-fruit wort in relation to classic honey wort, because of a wide range of organic acids in Cornelian cherry, such as: mallic acid, oxalic acid and citric acid. As presented in the results, the honey fruit wort also had a significantly higher sugar content. The higher content of sugars and organic acids created favorable conditions for higher HMF production in meads [38]. However, our research shows a positive change in mead production. No undesirable HMF was identified in young meads after alcoholic fermentation conducted with *Saccharomyces* yeast. The yeast probably utilized or degraded this compound metabolically. After three months of aging, HMF appeared in the final products, however, it was at least two times lower than in the wort, before fermentation and aging. It has also been shown that as a result of the alcoholic fermentation of honey wort, the acidity of classic young mead increased by 9.5%, while the acidity of meads with the addition of Cornelian cherry juice by an average of 5%. In all tested meads, the aging process increased the acidity of the finished products by an average of 2%.

The control of the carbohydrate profile during the ethanolic fermentation process is an important analysis that allows you to monitor the degree of extract consumption, which is influenced by the technological process and the type of substrates. What is more, both the sugar and alcohol content in the final products have an impact on the sweetness sensation in the meads, which directly shapes the sensory properties of these beverages [12,39]. Another essential ingredient of mead is glycerol, which also affects the sweetness, smoothness of the product or fullness of taste [39]. The acetic acid content in the meads was at the right level. Too high a content of this compound would be undesirable and could indicate that the yeast was exposed to osmotic stress [39]. Acidity is also an important quality determinant of fermented beverages, because it affects not only their taste but also microbiological stability. This parameter should be constantly monitored, as a sharp drop in the pH value may reduce the viability and vitality of the yeast [19]. HMF is also an important compound determinant of the quality of mead, because a sufficiently low concentration of HMF allows the production of high-quality safe products [40].

## 4. Conclusions

The process of mead production technology, which consists of the alcoholic fermentation stage and then the aging stage, has a significant impact on the change of antioxidant activity and the content of biologically active compounds in the final products. The antioxidant activity of classic and fruit meads can be reduced by up to 40%. Therefore, it is essential to conduct future research on the change of the antioxidant potential of these fermented beverages produced in various conditions of wort preparation, fermentation, aging and storage, to preserve as much natural antioxidants as possible. Apart from gallic and ellagic acids, the content of which increased up to four times as a result of the production of mead, the content of all other identified biologically active compounds (such as caffeoylquinic acids, anthocyanins, flavonols and iridoids) in the final products was reduced. It has been proved that 10% supplementation of mead with fruit juice with a high content of antioxidant compounds, such as Cornelian cherry juice, made it possible to obtain natural fermented beverages based on honey with almost 100 times higher antioxidant potential in relation to classic mead.

## Figures and Tables

**Table 1:** Basic physicochemical parameters of classic and Cornelian cherry meads.

Sample	Glucose	Fructose	Alcohol	Glycerol	Acetic Acid	HMF	pH
	g/L					mg/100 mL	
0W	134.34 ± 0.23 a ^1^	83.33 ± 0.44 a	na ^2^	nd ^3^	nd	1.62 ± 0.23 c	3.69 ± 0.01 a
0F	15.80 ± 0.46 c	37.35 ± 0.12 c	96.90 ± 0.75 d	7.65 ± 0.14 d	1.44 ± 0.00 a	nd	3.44 ± 0.00 b
0A	5.40 ± 0.54 d	15.88 ± 1.46 d	115.65 ± 0.65 a	8.15 ± 0.18 c	1.44 ± 0.01 a	0.33 ± 0.01 e	3.38 ± 0.00 c
JW	149.66 ± 0.10 a	95.70 ± 0.03 a	na	nd	nd	1.99 ± 0.09 b	3.46 ± 0.02 b
JF	28.20 ± 0.38 b	64.20 ± 0.22 b	92.90 ± 0.23 e	7.75 ± 0.09 d	1.06 ± 0.01 c	nd	3.25 ± 0.01 c
JA	25.80 ± 0.69 b	63.10 ± 0.61 b	103.20 ± 0.48 c	9.15 ± 0.36 b	1.10 ± 0.03 bc	1.21 ± 0.65 d	3.17 ± 0.00 cd
KW	157.77 ± 1.37 a	99.66 ± 1.04 a	na	nd	nd	1.98 ± 0.25 b	3.55 ± 0.01 ab
KF	30.00 ± 0.03 b	65.85 ± 0.46 b	91.60 ± 0.46 e	8.45 ± 0.44 c	1.05 ± 0.02 c	nd	3.35 ± 0.02 c
KA	27.40 ± 0.89 b	64.30 ± 0.02 b	104.50 ± 0.99 c	9.10 ± 0.32 b	1.13 ± 0.12 b	1.11 ± 0.66 d	3.32 ± 0.00 c
PW	154.14 ± 0.98 a	95.62 ± 0.40 a	na	nd	nd	2.17 ± 0.09 a	3.51 ± 0.00 ab
PF	32.25 ± 0.20 b	68.00 ± 1.30 b	89.90 ± 0.31 e	8.65 ± 0.09 c	1.07 ± 0.08 c	nd	3.34 ± 0.00 c
PA	27.80 ± 1.01 b	65.00 ± 0.89 b	107.30 ± 0.24 b	9.60 ± 0.13 a	1.17 ± 0.11 b	0.96 ± 0.19 b	3.23 ± 0.01 c

^1^ Values are expressed as the mean (*n* = 3) ± standard deviation. Mean values with different letters (a, b, c, etc.) within the same row are statistically different (*p*-value < 0.05).^2^ na, not applicable ^3^ nd, not detected.

## Data Availability

Not applicable.

## Supplementary Materials

The following are available online at https://www.mdpi.com/article/10.3390/biom11081113/s1, Figure S1: HPLC-PDA chromatograms (520 nm) of anthocyanins of fruit meads: Df-gal—delphinidin 3-*O*-galactoside; Cy-gal—cyanidin 3-*O*-galactoside; Cy-rob—cyanidin 3-*O*-robinobioside; Pg-gal—pelargonidin 3-*O*-galactoside; Pg-rob—pelargonidin 3-*O*-robinobioside, Figure S2: HPLC-PDA chromatograms (360 nm) of flavonols and ellagic acid of fruit meads: Ar-glc—aromadendrin 7-*O*-glucoside; EA—ellagic acid; Q-glcr—quercetin 3-*O*-glucuronide, Kf-gal—kaempferol 3-*O*-galactoside, Figure S3: HPLC-PDA chromatograms (320 nm) of phenolic acids of fruit meads: CQA—caffeoylquinic acids, Figure S4: HPLC-PDA chromatograms (280 nm) of phenolic acids of fruit meads: GA—gallic acid, Figure S5: HPLC-PDA chromatograms (245 nm) of iridoids of fruit meads: LA—loganic acid; L—loganine; S—sweroside; Co—cornuside, Table S1: Identification (UPLC retention times, spectra UV/Vis, and MS) of compounds of cornelian cherry juice by LC-MS.
