# Supplementary material for: Changes in the Antioxidative Activity and the Content of Phenolics and Iridoids during Fermentation and Aging of Natural Fruit Meads
Source: Biomolecules. 2021 Jul 28;11(8):1113. doi: 10.3390/biom11081113 (PMC8394733; doi:10.3390/biom11081113)
Supplement: Supplementary file 1 [file biomolecules-11-01113-s001.zip › biomolecules-1288841-supplementary.pdf]

Article

Changes in the Antioxidative Activity and the Content of Phenolics and Iridoids during Fermentation and Aging of Natural Fruit Meads

Kinga Adamenko [1,*], Joanna Kawa-Rygielska [1], Alicja Z. Kucharska [2], Adam Głowacki [1] and Narcyz Piórecki [3,4]

1 Department of Fermentation and Cereals Technology, Wrocław University of Environmental and Life Sciences, 51-630 Wrocław, Poland; joanna.kawa-rygielska@upwr.edu.pl (J.K.-R.); adam.glowacki@upwr.edu.pl (A.G.)
2 Vegetable and Plant Nutraceutical Technology, Department of Fruit, Wrocław University of Environmental and Life Sciences, 51-630 Wrocław, Poland; alicja.kucharska@upwr.edu.pl
3 Institute and Arboretum of Physiography in Bolestraszyce, 37-700 Przemyśl, Poland; narcyz360@gmail.com
4 Faculty of Physical Educaiton, University of Rzeszów, Towarnickiego 3, 35-959 Rzeszów, Poland
* Correspondence: kinga.adamenko@upwr.edu.pl; Tel.: +48-71-323-9418

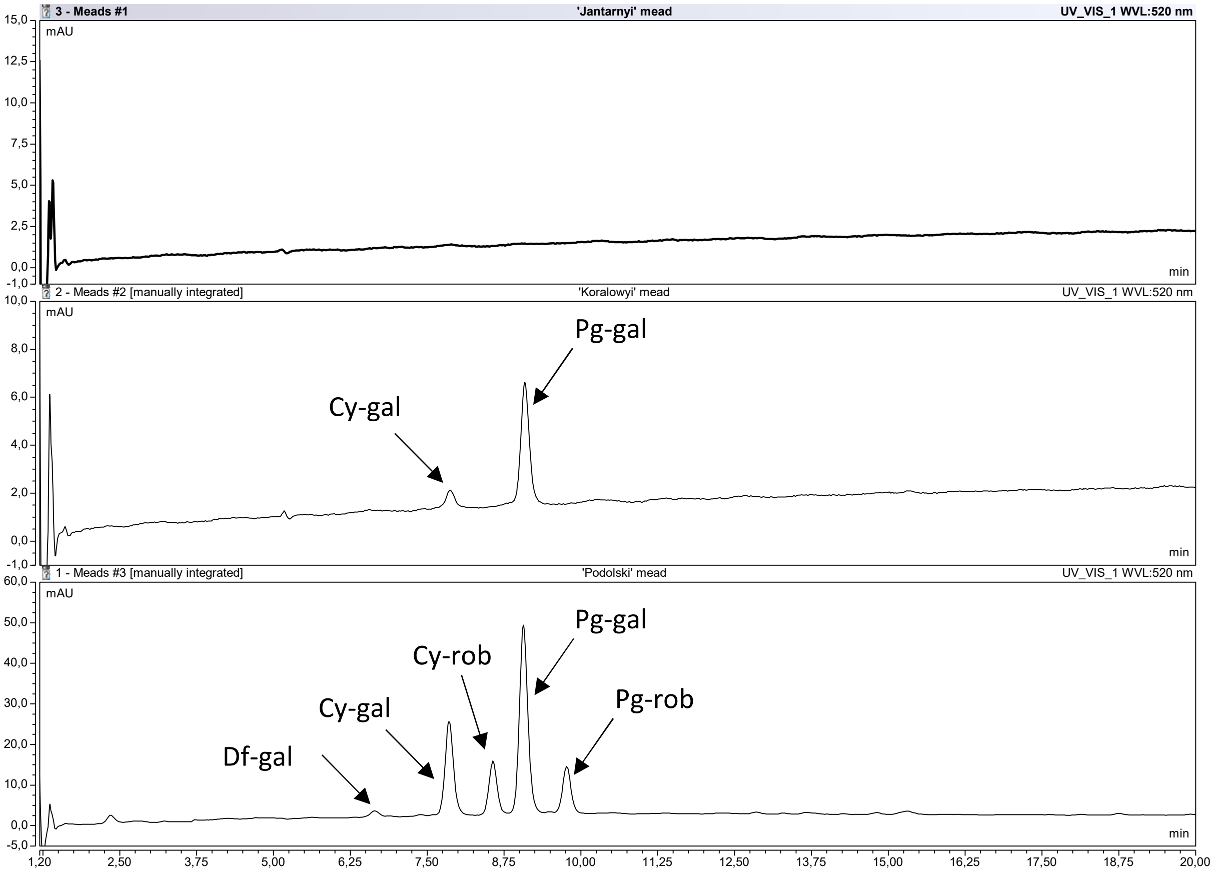

Figure S1. HPLC-PDA chromatograms (520 nm) of anthocyanins of fruit meads: **Df-gal**–delphinidin 3-*O*-galactoside; **Cy-gal**–cyanidin 3-*O*-galactoside; **Cy-rob**–cyanidin 3-*O*-robinobioside; **Pg-gal**–pelargonidin 3-*O*-galactoside; **Pg-rob**–pelargonidin 3-*O*-robinobioside

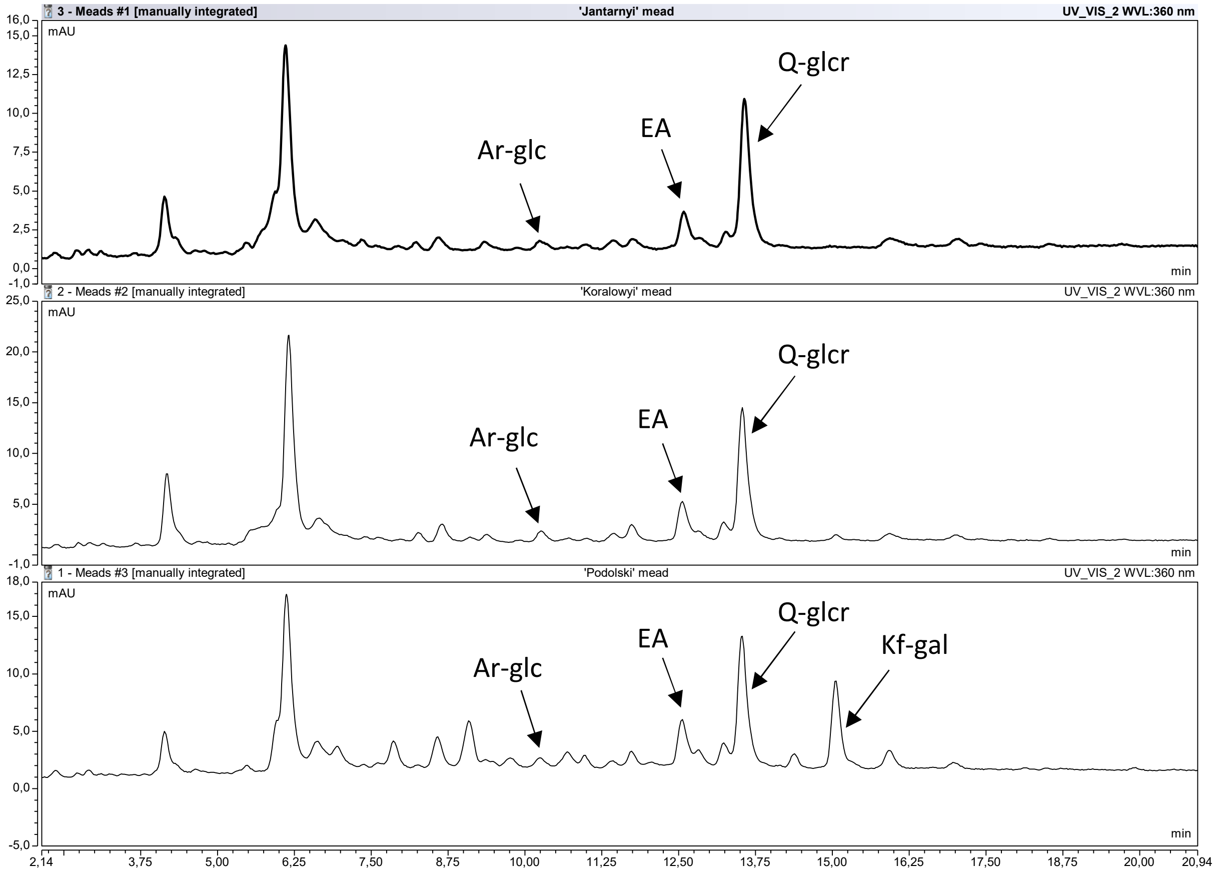

Figure S2. HPLC-PDA chromatograms (360 nm) of flavonols and ellagic acid of fruit meads: **Ar-glc**–aromadendrin 7-*O*-glucoside; **EA**–ellagic acid; **Q-glcr**–quercetin 3-*O*-glucuronide, **Kf-gal**–kaempferol 3-*O*-galactoside.

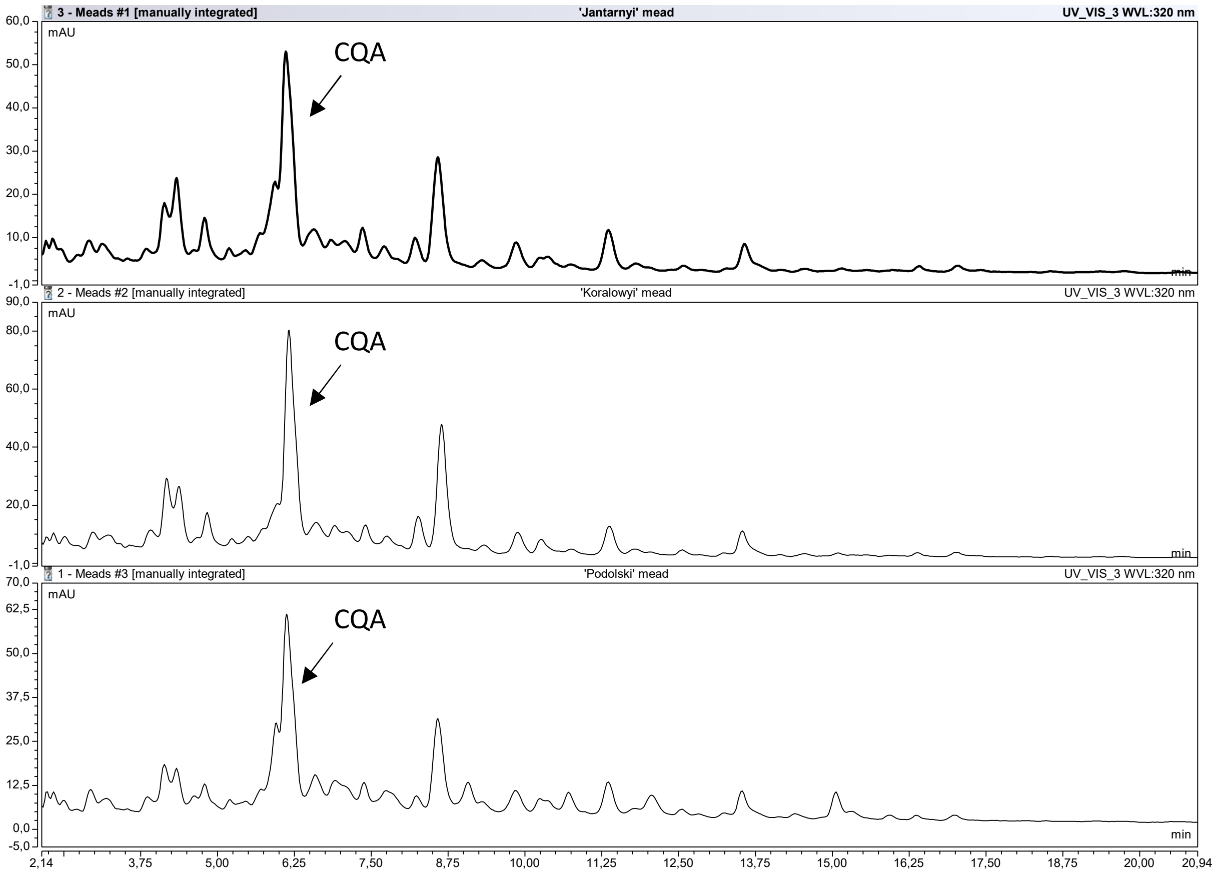

Figure S3. HPLC-PDA chromatograms (320 nm) of phenolic acids of fruit meads: **CQA**–caffeoylquinic acids.

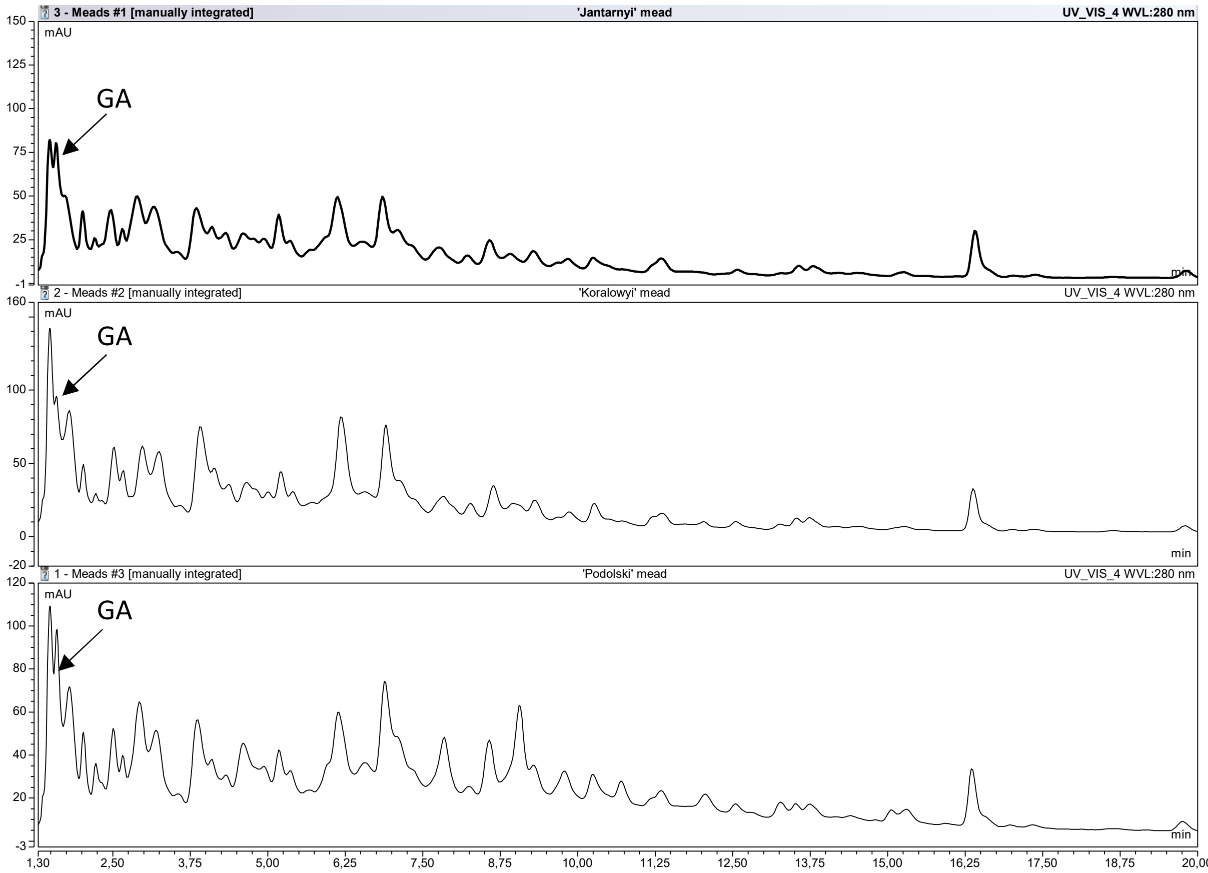

Figure S4. HPLC-PDA chromatograms (280 nm) of phenolic acids of fruit meads: **GA**–gallic acid.

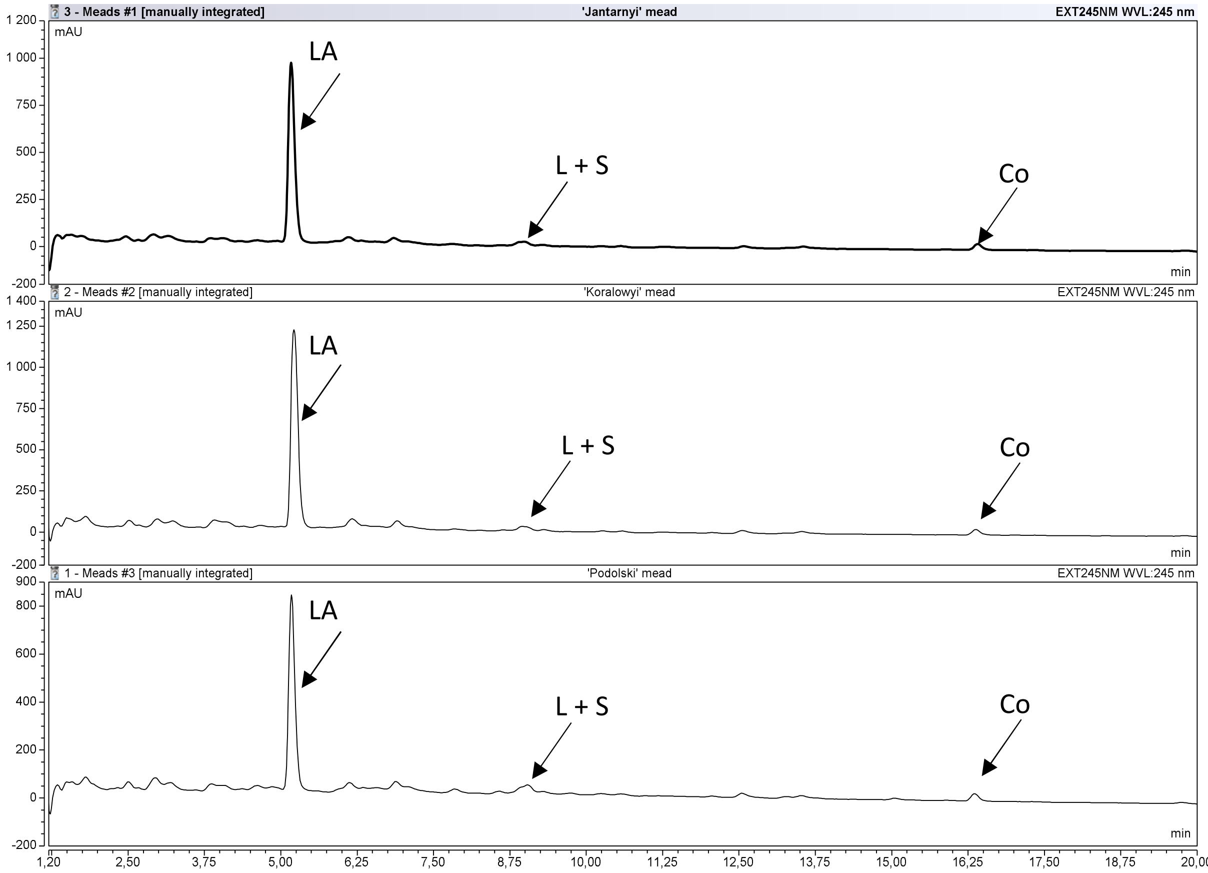

Figure S5. HPLC-PDA chromatograms (245 nm) of iridoids of fruit meads: **LA**–loganic acid; **L**–loganine; **S**–sweroside; **Co**–cornuside.

Table S1. Identification (UPLC retention times, spectra UV/Vis, and MS) of compounds of cornelian cherry juice by LC-MS.

Peak No.	$t_{R\ LC\text{-}MS}$ (min)	Compound	UV/Vis λ_{max} (nm)	$[M-H]^-/[M+H]^+$ (*m/z*)	Other ions (*m/z*)
1	3.7	Gallic acid	272	169	-
2	4.9	Loganic acid	246	375 (377^+)	213 (215^+)
3	5.8	Caffeoylquinic acid	324	353	191
4	5.73	Loganin	246	389	227<br>435 $[M + HCOO\text{-}H]^-$
5	5.73	Sweroside	246	357	195<br>403 $[M + HCOO\text{-}H]^-$
6	6.3	Delphinidin 3-*O*-galactoside	524	463^+	303^+
7	7.6	Cyanidin 3-*O*-galactoside;	515	449^+	287^+
8	8.3	Cyanidin 3-*O*-robinobioside	516	595^+	287^+
9	8.8	Pelargonidin 3-*O*-galactoside	501	433^+	271^+
10	9.5	Pelargonidin 3-*O*-robinobioside	501	579^+	271^+
11		Aromadendrin 7-*O*-glucoside	295	449	287
12	12.3	Ellagic acid	254/362	301	-
13	13.2	Quercetin 3-*O*-glucuronide	354	477	301
14	14.8	Kaempferol 3-*O*-galactoside	348	447	285
15	16.1	Cornuside	245/273	541 (543^+)	169 (171^+)

Identification of iridoids and polyphenols by LC-MS

The method was previously described by Kucharska et al. [2017]. Identification of compounds was performed using the Acquity ultra-performance liquid chromatography (UPLC) system, coupled with a quadrupole-time of flight (Q-TOF) MS instrument (UPLC/Synapt Q-TOF MS, Waters Corp., Milford, MA, USA), with an electrospray ionization (ESI) source. Separation was achieved on an Acquity BEH C18 column (100 mm × 2.1 mm i.d., 1.7 µm; Waters). The mobile phase was a mixture of 2.0% aq. formic acid *v*/*v* (A) and acetonitrile (B). The gradient program was as follows: initial conditions—1% B in A, 12 min—25% B in A, 12.5 min—100% B, 13.5 min—1% B in A. The flow rate was 0.45 mL/min and the injection volume was 5 L. The column was operated at 30 °C. UV–vis absorption spectra were recorded on-line during UPLC analysis, and the spectral measurements were made in the wavelength range of 200–600 nm, in steps of 2 nm. The major operating parameters for the Q-TOF MS were set as follows: capillary voltage 2.0 kV, cone voltage 40 V, cone gas flow of 11 L/h, collision energy 28–30 eV, source temperature 100 °C, desolvation temperature 250 °C, collision gas, argon; desolvation gas (nitrogen) flow rate, 600 L/h; data acquisition range, *m/z* 100–1000 Da; ionization mode, negative (iridoids, phenolic acids, flavonols) and positive (anthocyanins). The data were collected with Mass-LynxTM V 4.1 software (Waters Corp., Milford, MA, USA). The runs were monitored at the following wavelengths: iridoids at 245 nm, phenolic acids at 320 nm, 254 nm, 280 nm, flavonols at 360 nm, anthocyanins at 520 nm.

Kucharska, A.Z.; Sokół-Łętowska, A.; Oszmiański, J.; Piórecki, N.; Fecka, I. Iridoids, phenolic compounds and antioxidant activity of edible honeysuckle berries (*Lonicera caerulea* var. *kamtschatica Sevast.*). *Molecules* **2017**, *22*, 405–425.
